# Granular cell tumors: a report of six cases

**DOI:** 10.1186/1477-7819-10-204

**Published:** 2012-09-29

**Authors:** Kei Aoyama, Takako Kamio, Azusa Hirano, Akiyoshi Seshimo, Shingo Kameoka

**Affiliations:** 1 Department of Surgery II, Tokyo Women’s Medical University, 8-1, Kawada-cho, Shinjuku-ku, Tokyo, 162-8666, Japan

**Keywords:** Granular cell tumor

## Abstract

Granular cell tumor is a soft tissue neoplasm that originates in the nervous system and arises at virtually any body site, but is mainly found in the skin, oral cavity or digestive tract. Most are benign and reportedly malignant cases are rare, occurring in only 1% or 2% of cases. We report on our findings in six patients who developed granular cell tumor in the mammary gland, esophagus, subcutaneous tissue and muscle. Of six patients, two had granular cell tumor located in the breast, two in the submucosa of the esophagus, and the other two under the skin of the left axillary cavity and in the right latissimus dorsi muscle, respectively. One of the two patients with tumor in the submucosa of the esophagus also had esophageal cancer. Patients’ age ranged from 41 to 70 years (average, 59.1 years). Two patients with tumor in the submucosa of the esophagus were men, and the others were women. All of them were given a diagnosis of granular cell tumor by tissue biopsy and examination of excised specimens, but no evidence of malignancy was found. No recurrence has been noted in the patients who underwent surgical tumor removal.

## Background

Granular cell tumor is a soft tissue neoplasm that originates in the nervous system and arises at virtually any body site, but is mainly found in the skin, oral cavity or digestive tract [1-3]. Most of them are benign and reportedly malignant cases are rare, occurring in only 1% or 2% of cases [4-7]. We report on our findings in six patients who developed granular cell tumor in the mammary gland, esophagus, subcutaneous tissue and muscle.

## Case presentation

### Patient 1

**Figure 1:**
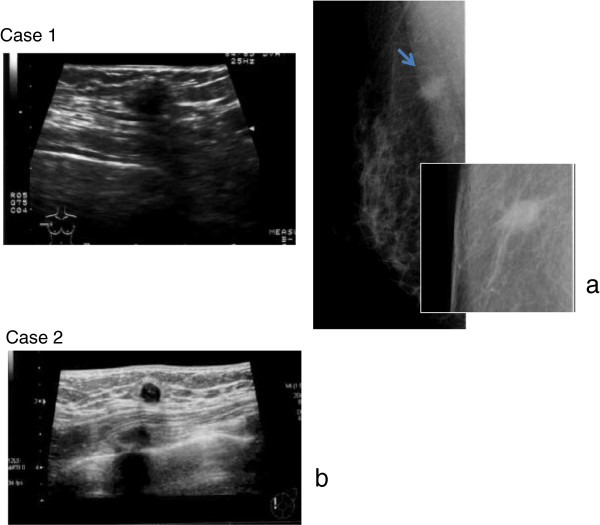
**(a) Case 1.** Ultrasonography revealed a tumor 8 × 9 mm in size with irregular borders and rupture of the anterior border of the mammary gland. Mammography revealed a tumor with irregular borders and spicule. (**b**) Case 2. Ultrasonography revealed a tumor 7 × 8 mm in size with irregular borders and rupture of the anterior border of the mammary gland.

**Figure 2:**
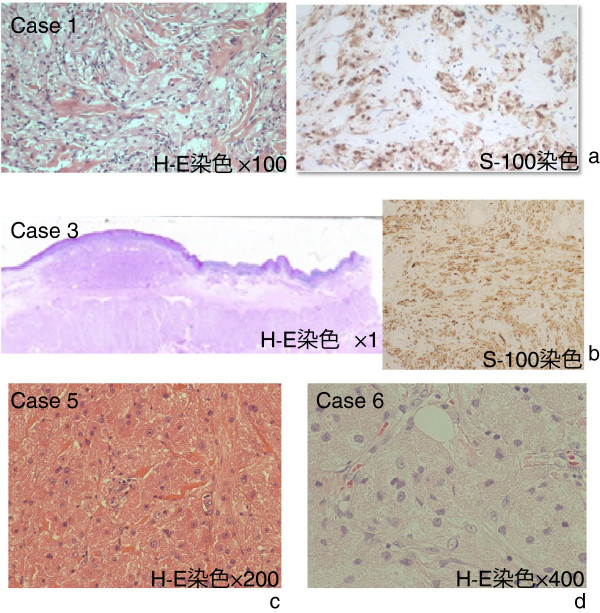
**(a) Case 1.** Histopathologic examination showed that the tumor cells had numerous granules, and immunostaining revealed S-100-positive (H&E staining, × 100). (**b**) Case 3. Histopathologic examination showed a submucosal tumor consisting of cells containing eosinophilic granules, and immunostaining revealed S-100-positive. The tumor was not a metastasis of esophageal cancer, and had no signs of malignancy (H&E staining, × 1). (**c**) Case 5. Histopathologic examination revealed a tumor in the striated muscular tissue. The tumor cells possessed numerous eosinophilic granules, and the tumor itself consisted of a dense proliferation of large tumor cells (H&E staining, × 200). **d**: Case 6. Histopathologic examination revealed eosinophilic granules inside the cell bodies. The cell nucleus was oval, and the nucleoplasmic ratio was low (H&E staining, × 400).

A 63-year-old woman. After an anomaly was detected by mammography (MMG) in a medical check-up at a hospital, she was referred to our hospital for further examination. No major diseases were recorded in her past history. By palpation, a tumor with good motility and a smooth surface was felt on the outer quadrant of the right breast. MMG revealed a tumor shadow with spicule in the outer right part, and ultrasonography (US) showed an irregular shaped tumor in the right C region (Figure 1a). Needle tissue biopsy did not reveal malignant cells, but both MMG and US yielded findings indicating the presence of breast cancer (invasive ductal carcinoma); therefore, lumpectomy was performed. Histopathologic examination confirmed the diagnosis of granular cell tumor (Figure 2a).

### Patient 2

A 41-year-old woman. After an anomaly was detected by MMG in a medical check-up at a hospital, she was referred to our hospital for further examination. No major diseases were recorded in her past history. By palpation, an induration was felt on the inner upper quadrant of the left breast. US revealed an irregular shaped tumor in the left A region (Figure 1b). Because the images suggested the presence of breast cancer, needle tissue biopsy was performed. Histopathologic examination revealed granular cell tumor.

### Patient 3

**Figure 3:**
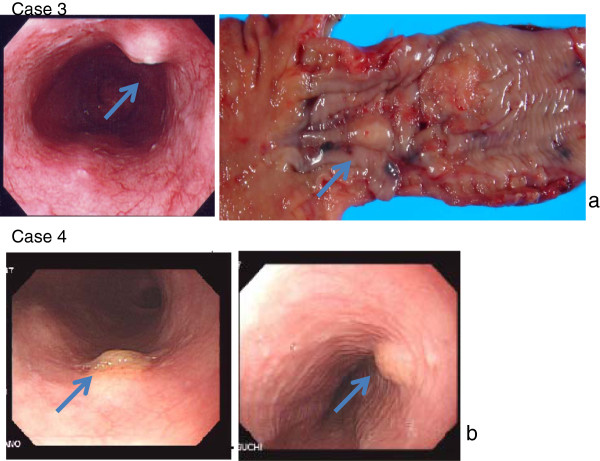
**(a) Case 3.** Upper gastrointestinal endoscopy suggested esophageal cancer, and revealed a submucosal tumor of yellowish white at a position below the cancer. Total thoracic esophagectomy was performed and the submucosal tumor was also removed as a combined resection. The macroscopic diameter of the submucosal tumor was about 9 mm. (**b**) Case 4. Upper gastrointestinal endoscopy performed for a screening purpose revealed a trapezoidal protruded lesion of yellowish-white at a position 30 cm from the incisor tooth.

A 70-year-old man came to our hospital to have surgery for esophageal cancer and underwent detailed examination. There were no significant diseases or symptoms in his medical history. Upper gastrointestinal endoscopy revealed a submucosal tumor (SMT) at a position below the esophageal cancer lesion (Figure 3a). Histopathologic examination of the resected specimen after esophageal cancer surgery yielded a diagnosis of granular cell tumor (Figure 2b).

### Patient 4

A 67-year-old man. Upper gastrointestinal endoscopy showed a SMT in the esophagus at a position about 30 cm from the incisor tooth (Figure 3b), and tissue biopsy was performed. Histopathologic examination yielded a diagnosis of granular cell tumor.

### Patient 5

**Figure 4:**
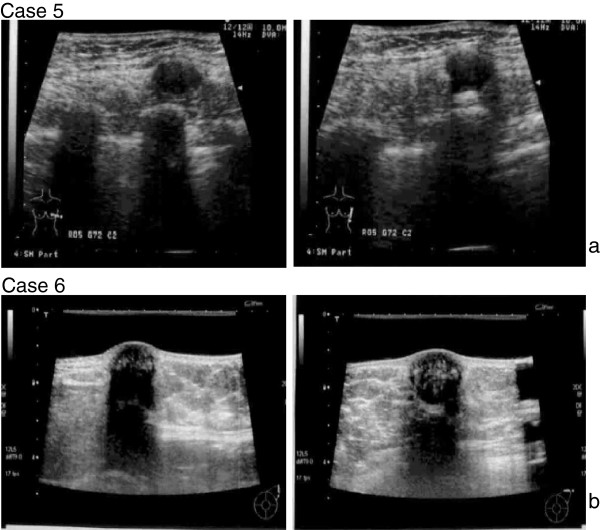
**(a) Case 5.** Ultrasonography revealed an irregularly bordered, low echoic tumor that protruded in a convex shape from the intrinsic muscle layer of the latissimus dorsi muscle. The size was 10 × 14 mm. (**b**) Case 6. Ultrasonography revealed a smoothly bordered tumor with posterior echo attenuation, having heterogeneous content and 18 × 17 mm in size.

A 60-year-old woman felt a tumor-like lump in the anterior axillary line outside of the right breast, and visited our hospital. MMG and US did not disclose obvious evidence of tumor in the breast, but revealed an oval-shaped, low-echoic tumor in the latissimus dorsi muscle (Figure 4a). Histopathologic examination after lumpectomy yielded a diagnosis of granular cell tumor (Figure 2c).

### Patient 6

A 54-year-old woman felt a tumor-like lump in the left axillary cavity, and visited our hospital. US revealed an oval-shaped, low-echoic tumor under the skin in the left axillary region (Figure 4b). Lumpectomy was performed and granular cell tumor was diagnosed by postoperative histopathologic examination (Figure 2d).

Of six patients, two had a granular cell tumor located in the breast, two in the submucosa of the esophagus, one under the skin of the left axilla and one within the right latissimus dorsi muscle. One of the two patients having a tumor in the submucosa of the esophagus also had esophageal cancer. The patients’ age ranged from 41 to 70 years (average, 59.1 years). Two patients having a tumor in the submucosa of the esophagus were men, and the others were women. All of them were given a diagnosis of granular cell tumor by needle tissue biopsy and biopsy of excised specimens, but no evidence of malignancy was found (Figure 2a-d). No recurrence has been noted in patients who underwent surgical tumor removal (Table 1).

**Table 1:** **Clinicopathologic findings in granular cell tumor cases**

**Case**	**Age**	**Sex**	**Site**	**Diameter**	**Diagnosis**
1	63	Female	Mammary gland	9 mm	Benign
2	41	Female	Mammary gland	8 mm	Benign
3	70	Male	Esophagus	9 mm	Benign
4	67	Male	Esophagus	6 mm	Benign
5	60	Female	Skeletal muscle (latissimus dorsi muscle)	14 mm	Benign
6	54	Female	Axillary cavity (subcutaneous tissue)	18 mm	Benign

A granular cell tumor that has occurred in the breast is often diagnosed to be invasive ductal carcinoma by imaging diagnostic techniques, and patients in such a case showed spicule on MMG and an irregular shaped tumor on US. A granular cell tumor that has developed in the submucosa of the esophagus usually appears as a non-pedunculated SMT with a smooth surface and pale yellow color, and both of our two cases presented the same characteristics. The incidence of granular cell tumor in the skin is high, but it is very rare for it to develop in the skeletal muscle. One of our patients was found with a granular cell tumor in the skeletal muscle (latissimus dorsi muscle) (Table 1).

## Discussion

Granular cell tumor was first reported by Abrikossoff [1] by the name of granular cell myoblastoma. One of its characteristics is that eosinophilic granules are contained in the cytoplasm of cells. Since the positive rates for S-100 protein and neuron specific enolase are high, currently it is thought that this tumor originates from Schwann cells. They account for an incidence of 0.5% among soft tissue tumors, and it has been reported that they primarily occur in or beneath the skin, or in the submucosa of the tongue, throat, chest wall, or bronchus. The tumor is commonly found in those aged 10 to 50 years old, and more often in women than in men [3,8-11]. Granular cell tumor of the breast is largely a disease affecting women, as it is with other breast malignancies, but has also been described in men, accounting for 6.6% of granular cell tumor of the breast [12].

About 2% of all granular cell tumor cases are malignant [4-7]. A tumor 3 cm or less in size can be regarded as benign. However, if the tumor grows rapidly and forms an ulcer, malignancy should be suspected.

From a histopathologic perspective, Fanburg Smith and colleagues [5] proposed the following six criteria to determine whether a tumor is malignant or not: (1) the presence of necrosis, (2) the emergence of spindle cells, (3) a vacuolar nucleus with an enlarged nuclear body, (4) increase in nuclear division (2 mitoses/10HPF), (5) increase in the nucleoplasmic ratio, and (6) polymorphism. If none of these diagnostic criteria are met, the tumor is considered to be benign. If one or two criteria are met, the tumor is considered to be atypical, and if three or more criteria are met, the tumor is considered to be malignant.

Sonobe and colleagues [6] divided malignant granular cell tumors into two groups: those that are malignant both histologically and clinically, and those that are histologically benign but clinically malignant. The most common metastasis sites of malignant granular cell tumor are the lymph nodes, followed by the lungs. Insufficient tumor resection often results in local recurrence, and has a tendency to spread both lymphogenously and hematogenously. Reportedly, chemotherapy and radiotherapy treatments cannot be expected to be effective, with surgical resection being the primary option [4-7]. Resection with adequate margins is necessary because the tumor has no capsule and is proliferation invasive. All tumors in our six cases were small in diameter, and benign histologically (Table 1).

In many cases, a granular cell tumor that has occurred in the breast is diagnosed to be invasive ductal carcinoma by imaging, and our patients with such a condition also showed spiculation on MMG images [13-18]. The MMG and US appearances of granular cell tumor pose a diagnostic dilemma because of its similarity to breast malignancy.

A granular cell tumor that has developed in the submucosa of the esophagus is a non-pedunculated SMT with a smooth surface and pale yellow color, and both of our two cases presented the same characteristics [19-22]. Granular cell is uncommon, mainly occurring on the skin, tongue and oral cavity as a single nodule. The disease in 30% to 45% of cases affects the skin, followed by the area of the head and neck, where the most frequent location is intraoral in the tongue and the soft and hard palate [11,19-24]. Other locations affected are the breast, the gastrointestinal tract, the respiratory tract, the thyroid gland, the urinary bladder, the central nervous system, and female genitalia. Location in the skeletal muscle region, as in our case, is rare. One of our patients was found with a granular cell tumor in the skeletal muscle [25].

In the gastrointestinal tract, the tumor often occurs in the esophagus and is rarely found in the large intestine [19-22]. Granular cell tumor that has developed in the gastrointestinal tract must be differentiated from other submucosal tumors such as steatoma, smooth muscle tumor, neurogenic tumor and gastrointestinal stromal tumor. On endoscopic examination, the tumor is a very hard, smooth-surfaced submucosal tumor which appears as a yellow or yellowish-white hemispherical protrusion with a thin mucous membrane, and is sometimes called "molar tooth" or "sweet corn." It is rare that ulcerous lesions or recesses are observed on the mucosal surface [19-22].

For differential diagnosis of a granular cell tumor in the subcutaneous tissue and muscle, candidates include: malignant fibrous histiocytoma, alveolar soft part sarcoma, desmoid, granulomatous, and nodular fasciitis [7,23-25]. Granular cell tumor of the breast arises from intralobular breast stroma and occurs within the distribution of the cutaneous branches of the supraclavicular nerve [17,18]. When occurring in the breast, the tumor is often found in the upper inner quadrant unlike breast cancer that is found in the upper outer quadrant [17,18]. Since the tumor affects the innervation of the skin, contractions or shrinkage of the skin sometimes occur. Traditional imaging techniques, including MMG and US scanning, are widely employed when investigating the presence mass. However, the issue with these modalities in cases of granular cell tumor is that the radiological findings are often indistinguishable from breast cancer. On MMG, the tumor is seen as a substantial round-shaped lesion with distinct edges of the hyperplasia invading into the surrounding tissues, and irregularity, speculation, isodensity sometimes associated with hypodense rims, and heterogenicity are commonly observed (Figure 1). Mass calcification was not observed in our cases. On the other hand, on US, the edges of the hyperplasia are not distinct and attenuation of posterior echo often occurs. Common features include solid, heterogeneous, poorly defined masses with high depth/width ratio (Figure 1). They are generally hypo-echoic and display posterior shadowing with a coarse internal echo and high boundary echo. For these reasons, it is necessary to perform differential diagnosis between breast cancer and invasive ductal carcinoma based on the images [13-18]. The range of US findings are as broad as those of MMG, they are often suggestive of malignancy and most importantly there are no recognized features specific for granular cell tumor of the breast.

## Conclusion

Granular cell tumor is a neoplasm that develops in the soft tissues, mainly in the skin, oral cavity and gastrointestinal tract, but the tumor is relatively rare. Granular cell tumor in all of our cases was benign, and no recurrence after resection has been found.

## Consent

In accordance with the regulations of the Human Investigation Committee of the Tokyo Women’s Medical University, written informed consent was obtained from the patient for publication of this report and any accompanying images.

## Abbreviations

MMG: mammography; SMT: submucosal tumor; US: ultrasonography.

## Competing interest

The authors declare no conflicts of interest with respect to authorship and/ or publication of this article.

## Authors’ contributions

KA designed the study, researched the literature, and drafted the manuscript. TK, AH, AS, and SK participated in the study design and coordination, and helped to collect data. All authors have read and approved the manuscript.

## Funding

No extramural funding was used for this study.
